# Contemporary applications of vibrational spectroscopy in plant stresses and phenotyping

**DOI:** 10.3389/fpls.2024.1411859

**Published:** 2024-09-13

**Authors:** Isaac D. Juárez, Dmitry Kurouski

**Affiliations:** ^1^ Department of Biochemistry and Biophysics, Texas A&M University, College Station, TX, United States; ^2^ Interdisciplinary Faculty of Toxicology, Texas A&M University, College Station, TX, United States

**Keywords:** digital farming, non-invasive phenotyping, nutrient content assessment, plant disease diagnostics, Raman spectroscopy, optical sensing, infrared spectroscopy, surface enhanced Raman spectroscopy

## Abstract

Plant pathogens, including viruses, bacteria, and fungi, cause massive crop losses around the world. Abiotic stresses, such as drought, salinity and nutritional deficiencies are even more detrimental. Timely diagnostics of plant diseases and abiotic stresses can be used to provide site- and doze-specific treatment of plants. In addition to the direct economic impact, this “smart agriculture” can help minimizing the effect of farming on the environment. Mounting evidence demonstrates that vibrational spectroscopy, which includes Raman (RS) and infrared spectroscopies (IR), can be used to detect and identify biotic and abiotic stresses in plants. These findings indicate that RS and IR can be used for in-field surveillance of the plant health. Surface-enhanced RS (SERS) has also been used for direct detection of plant stressors, offering advantages over traditional spectroscopies. Finally, all three of these technologies have applications in phenotyping and studying composition of crops. Such non-invasive, non-destructive, and chemical-free diagnostics is set to revolutionize crop agriculture globally. This review critically discusses the most recent findings of RS-based sensing of biotic and abiotic stresses, as well as the use of RS for nutritional analysis of foods.

## 1 Introduction

Most of economically important plants, such as corn, wheat and rice, can be infected with a large number of pathogens. Although a progression of plant diseases directly depends on the pathogen and weather conditions, in most cases, infected plants will decay within several weeks. This process can be decelerated if plant protection chemistry is timely utilized. Satellite- or drone-based RGB imaging can be used to identify such problem areas. However, these techniques lack specificity since the diagnostics is based on the color change. Both of these RGB images are laborious and expensive. These and other factors largely limit their broad application in modern farming. To overcome the lack of specificity, several molecular methods, such as PCR and ELISA, can be used. These methods directly rely on the presence of the pathogen in the analyzed sample. Although provide very high sensitivity and specificity, both methods are highly laborious, which limits cost-per-sample minimization. On average, one ELISA sample cost around $15, whereas one PCR test is ~$25. The major drawback of both methods is false-negative outcomes in the case of a lack of a pathogen or a pathogen nucleic acid in the sample. For instance, PCR can be efficiently used to diagnose citrus greening disease, also known as Huanglongbing (HLB) (Sanchez et al., 2019b). This disease is caused by bacteria that infect citrus trees. Infected trees exhibit chlorosis and premature fruit drop. Since the bacteria are vectored by psyllids, leaves in once tree branch may possess the pathogen, whereas leaves of the next branch on the same tree will be pathogen-free. In the former case, PCR provides a confirmatory pathogen identification. However, in the latter case, false-positive results will be delivered by this molecular assay. Furthermore, in hot summer seasons, bacteria move to the stem and roots of the trees. Consequently, analysis of plant leaves in these months will indicate falls pathogen-free status of the plants.

Abiotic stresses, such as drought, salinity, and heat, are far more detrimental to the crop yield. On average, these stresses are accountable for ~70% of the crop losses worldwide. Their diagnostic is far more challenging than the detection and identification of biotic stresses. Primarily because both PCR and ELISA cannot be used in such cases. RGB-based imaging is also limited because visual symptoms of biotic and abiotic stresses are very similar. Traditionally, induced coupled plasma mass spectroscopy (ICP-MS) is used to quantify macro- and micronutrients in both soil and plants. This information can be used to alter the dosage of plant fertilizers to mitigate abiotic stresses caused by the lack of nutrients. ICP-MS can be also used to probe plant contamination with heavy and toxic metals, such as lead and arsenic. However, ICP-MS is not portable, which requires sample shipment to analytical laboratories. This technique is also laborious and expensive.

**Figure 1:**
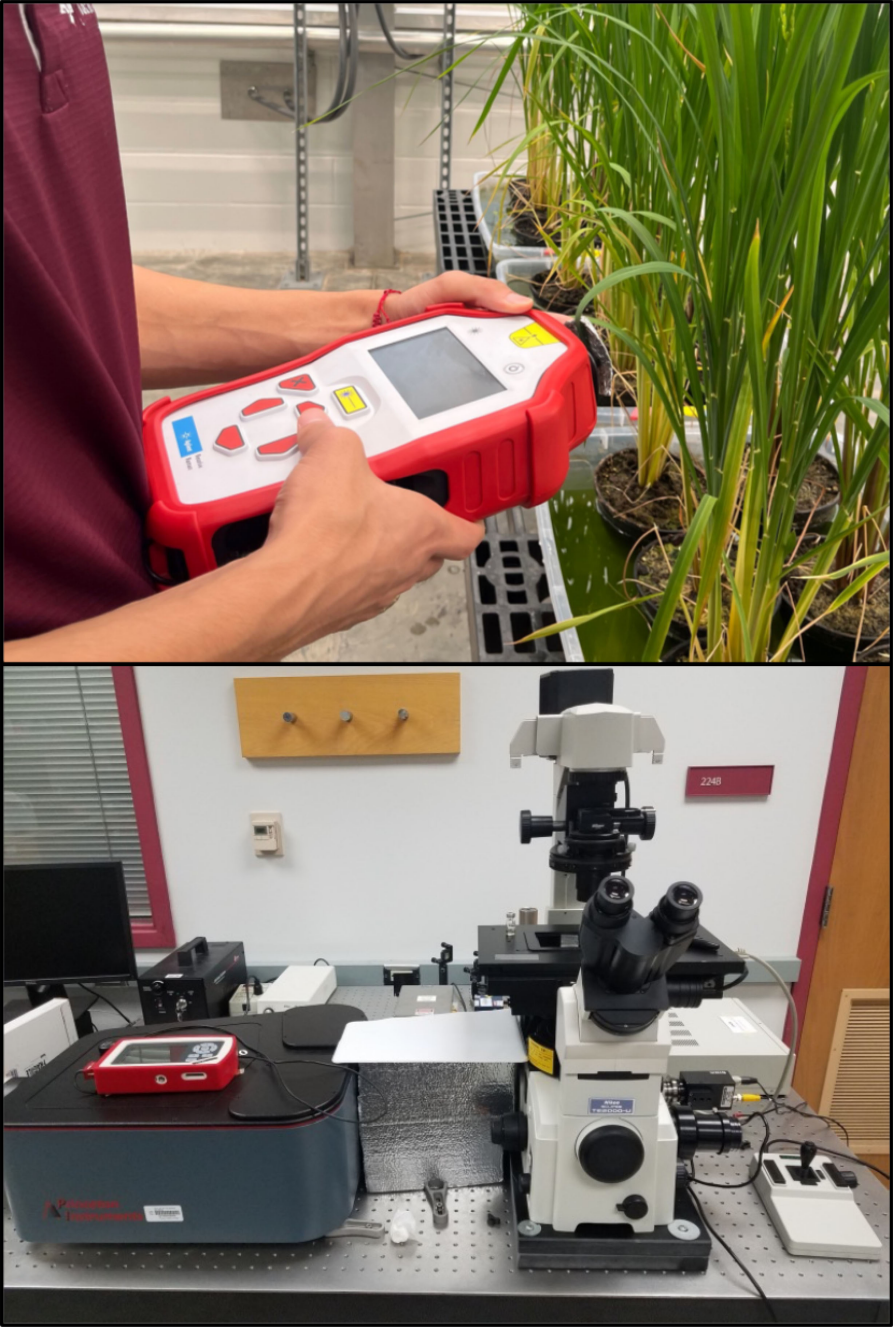
Commercially available hand-held Raman spectrometer with 830 nm excitations (top) and a bench-top home-built confocal Raman microscope (bottom).

One can expect that timely diagnostics of both biotic and abiotic stresses require new techniques that must be 1) unexpensive; 2) portable, 3) fast, and 4) accurate. During the past years, a growing number of studies demonstrated that both IR and RS fit these strict requirements. IR and RS are label-free methods that use light to probe the chemical structure of analyzed samples. Therefore, the direct cost of both IR- and RS-based analyses is zero. Several companies came to the market with excellent hand-held IR and RS instruments, **Figure 1**. Although their costs remain high ($20,000-$70,000), these instruments are easy to use. Furthermore, the direct time of spectral acquisition is typically around 1-2 s. In most cases, 10-20 s are required to process the data. Since most of the spectrometers are equipped with a display and a chemometric algorithms, the researcher can see the outcome of the spectral analysis within 15-25 s. The question remains unclear is whether such instruments can be used for an accurate, robust and reliable diagnostics of biotic and abiotic stresses in plants.

Robust and reliable plant phenotyping is highly important for plant breeders. Currently, such expertise requires years of training and experience. Since different plant species and plant varieties have distinctly different biochemical profiles, one can expect that RS could be used to detect these biochemical differences and, consequently, assist in plant breading.

This review will critically discuss the most recent reports on IR- and RS-based diagnostics of fungal, viral, and bacterial diseases, as well as on the use of both techniques for the quality control of fruits and vegetables. We also briefly discuss the most recent advances in RS-based plant breeding, prediction of the optimal harvest date and genotyping.

## 2 Instrumentation and imaging approaches

### 2.1 Raman spectroscopy

**Figure 2:**
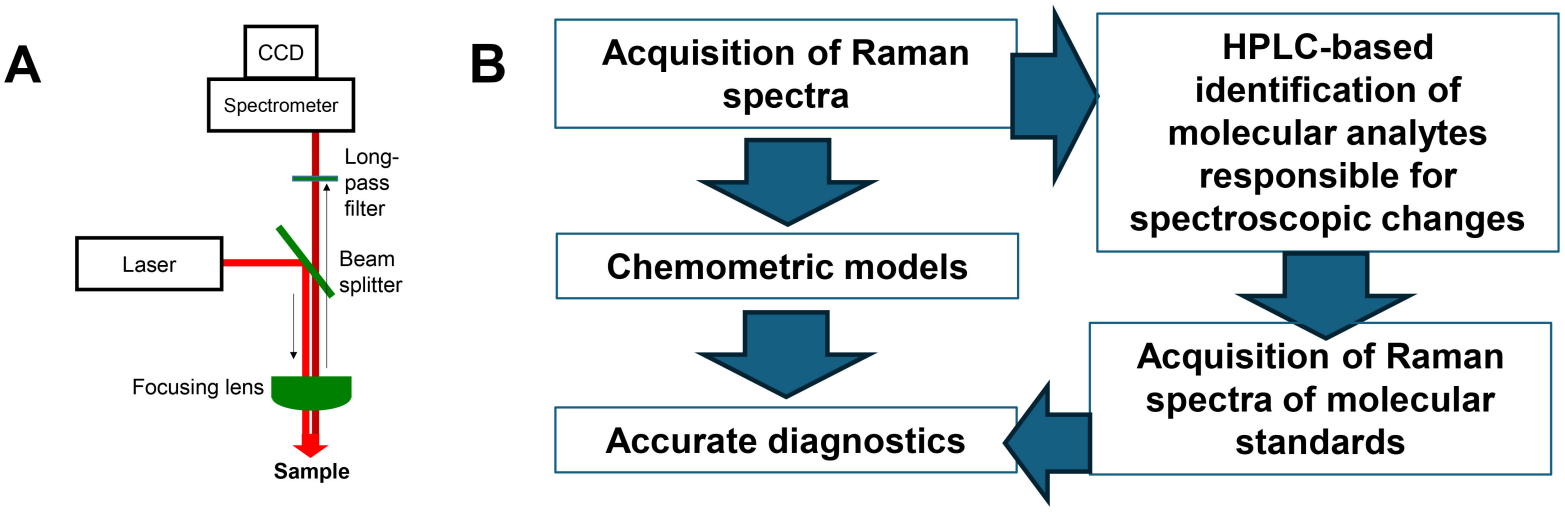
Schematic representation of **(A)** Raman spectrometer and **(B)** workflow of used to develop robust and reliable diagnostics of biotic and abiotic stresses in plants.

Both hand-held and bench-top Raman spectrometers share a very similar engineering concept. In most cases, continuous wavelength (CW) lasers are used to generate light that is directed towards a beam splitter by a set of mirrors, **Figure 2A**. Next, the light is focused on the sample either by a simple achromatic lens or by a microscope objective. The scattered light is collected by the same optical setup; Long-pass filter is then used to cut off elastically scattered light, whereas inelastically scattered photons are directed towards a spectrograph where they are split on a grating based on their energies. Finally, a CCD camera is used to collect the inelastically scattered photons. Any Raman spectrometer has several critically important parameters such as laser power, excitation wavelength, laser spot size and a spectral resolution. The first and second parameters are determined by the laser. Although currently available lasers can generate light with pretty much any wavelength ranging from deep UV (~190 nm) to far IR (1064 nm), electromagnetic radiation in the visible areas of the light spectrum is most commonly used. Our group showed that the use of blue and green light in Raman spectroscopy provided the advantage in the detection of carotenoids due to the resonance Raman effect. Plants possess ~20 different carotenoids with have slightly different Raman spectra. Thus, Raman spectrometers with blue and green laser sources provide the advantage of sensing these biological molecules in the plant leaves. We also showed that yellow and red lasers are not useful in optical sensing of plants due to a high chlorophyll fluorescence in this part of electromagnetic spectrum. At the same time, near IR lasers (785 nm, 830 nm and 1064 nm) can be used to overcome this limitation. Utilization of these lasers allows for sensing of a large number of biological molecules in plant samples. It should be noted that silicon-based CCD cameras cannot be used for collection of phonons with wavelength above 1.4 eV (880 nm). To overcome this limitation, InGaAs CCDs are used in the instruments with 1064 nm excitation. However, photon-to-electron conversion on these CCDs is not as good as in silicon-based CCDs. These heterostructure-based detectors also have much greater dark current noise, which results in much more nosier spectra compared to those acquired on silicon-based CCDs.

### 2.2 Surface-enhanced Raman spectroscopy

SERS is based on a phenomenon of strong (10^6^-10^8^) amplification of Raman scattering by metal nanostructures. If illuminated by light at or around their absorption maxima, metal nanostructures exhibit coherent oscillations of conductive electrons, also known as localized surface plasmon resonances (LSPRs). LSPRs enhance Raman scattering from molecules located in the close proximity to the metal surfaces allowing for single-molecule detection. Over the past decade, a large number of synthetic appraisals have been reported which could be used to fabricate nanostructures with the desired optical properties. Furthermore, nanostructures can be decorated with molecular analytes, also known as capture layer, to enable targeted sensing of the molecular species of interest. Although silver nanoparticles exhibit high cell toxicity, gold nanoparticles are proudly utilized for SERS sensing in life systems. Several groups demonstrated that utilization of copper or magnesium for the nanostructure fabrication allows for minimization of costs of the final product of syntheses.

### 2.3 Infrared spectroscopy

Rapid development of mid-IR and QCL lasers allowed for a substantial minimization of the IR spectrometers. In this instruments, IR light (3-13 μm) is directed to the interferometer to enable a Fourier transformed spectral acquisition. Next, the IR light is directed towards the sample (transmittance IR) or a crystal (attenuated total reflectance (ATR-IR). Finally, a MCT detector is used to collect the IR light. In the case of transmittance IR instruments, calcium fluoride or similar substrates are used as a sample support. In ATR-based IR systems, the sample can be directly pressed against the crystal for spectral analysis. If transmittance IR instruments require transparent or translucent samples, ATR-IR based setups can be used to analyze any type of the sample. ATR modality also allows for the development of hand-held IR instruments that can be used directly in the field. It should be noted that IR can be also coupled with atomic force microscopy (AFM). This instrumental setup is known as AFM-IR or photothermal IR. AFM-IR offers ~2-4 nm spatial resolution, which can be highly beneficial in the analysis of biological molecules, and single molecule sensitivity which cannot be achieved using conventional IR instruments.

It is important to emphasize that a term “near-IR spectroscopy” is commonly used to describe electronic absorbance or reflectance spectroscopy. In this case, electronic rather than vibronic properties of samples are probed.

### 2.4 Spectral analysis and interpretation

In the X axis of Raman spectra, a “Raman Shift” is used to describe the energy change between the incident and acquired inelastically scattered photons. Therefore, Raman spectra collected with different excitations will have the same spectra (except resonance Raman). Vibrations of a vast majority of biological molecules are in 300-1800 cm^-1^, as well as 2,200-3,500 cm^-1^. Since 2,200-3,500 cm^-1^ spectral window is primarily dominated by CH, CH_2_ and OH vibrations, most of the reported Raman spectra are within 300-1800 cm^-1^. The Y axis reflects the intensity of inelastically scattered photons. Spectral intensity is primarily dependent on the laser power and spectral acquisition time. Therefore, intensity in counts over laser power (mW) and seconds (s) can be used to describe the intensity of the acquired spectra. In IR spectra, a change in the absorbance intensity over a certain wavenumber is reported. Although less common, a change in transmittance over a certain wavenumber can be presented. It is important to remember that a relative intensity of bands in the spectra acquired in the transmittance and ATR modalities will change. Therefore, ATR correction should be applied to all IR spectra recorded in ATR mode.

If the overall IR spectral intensity directly depends on the amount of the material, intensity of Raman spectra depends on two factors: sample color and Raman cross-section. Since Raman is a scattering phenomenon, the darker is the sample color, the less intense Raman spectrum will be produced. Therefore, if the same type of materials with different colors, such as corn kernels or plant leaves, are analyzed, spectral normalization should be performed. Our group proposed to use 1440 and 1458 cm^-1^ bands for the spectral normalization. These bands originate from aliphatic (CH_2_) vibrations that present in nearly all classes of biological molecules. Therefore, this type of normalization is least biased for a comparison of intensities of vibrational bands that originate from biologically important molecules, such as carotenoids or sugars. Raman cross-section directly depends on the excitation wavelength. Since Raman scattering depends on the fourth power of the frequency of light, utilization of UV or near-UV light is far more beneficial compared to the IR or near IR light.

Interpretation of vibrational bands in the IR and Raman spectra of plant material is a challenging process. In the Raman spectra collected form plant leaves, vibrational bands originating from pectin, cellulose, phenylpropanoids, proteins, and carotenoids can be detected, **Tables 1**, **2**.

**Table 1:** Vibrational bands and their assignments for the Raman spectra collected from plant leaves and seeds.

Band (cm-1)	Vibrational mode	Assignment
480	C-C-O and C-C-C Deformations; Related to glycosidic ring skeletal deformations δ(C-C-C)+τ(C-O) Scissoring of C-C-C and out-of-plane bending of C-O	Carbohydrates (Almeida et al., 2010)
520	ν(C-O-C) Glycosidic	Cellulose (Edwards et al., 1997; Pan et al., 2017)
747	γ(C–O-H) of COOH	Pectin (Synytsya et al., 2003)
849-853	(C_6_–C_5_–O_5_–C_1_–O_1_)	Pectin (Engelsen and Nørgaard, 1996)
917	ν(C-O-C) In plane, symmetric	Cellulose, phenylpropanoids (Edwards et al., 1997)
964-969	δ(CH_2_)	Aliphatics (Yu et al., 2007; Cabrales et al., 2014)
1000-1005	In-plane CH_3_ rocking of polyene aromatic ring of phenylalanine	Carotenoids (Schulz et al., 2005); protein
1048	ν(C-O)+ν(C-C)+δ(C-O-H)	Cellulose, phenylpropanoids (Edwards et al., 1997)
1080	ν(C-O)+ν(C-C)+δ(C-O-H)	Carbohydrates (Almeida et al., 2010)
1115-1119	Sym ν(C-O-C), C-O-H bending	Cellulose (Edwards et al., 1997)
1155	C-C Stretching; v(C-O-C), v(C-C) in glycosidic linkages, asymmetric ring breathing	Carotenoids (Schulz et al., 2005),carbohydrates (Wiercigroch et al., 2017)
1185	ν(C-O-H) Next to aromatic ring+σ(CH)	Carotenoids (Schulz et al., 2005)
1218	δ(C-C-H)	Carotenoids (Schulz et al., 2005), xylan (Agarwal, 2014)
1265	Guaiacyl ring breathing, C-O stretching (aromatic); -C=C-	Phenylpropanoids (Cao et al., 2006), unsaturated fatty acids (Jamieson et al., 2018)
1286	δ(C-C-H)	Aliphatics (Yu et al., 2007)
1301	δ(C-C-H)+δ(O-C-H)+δ(C-O-H)	Carbohydrates (Cael et al., 1975; Almeida et al., 2010)
1327	δCH_2_ Bending	Aliphatics, cellulose, phenylpropanoids (Edwards et al., 1997)
1339	ν(C-O); δ(C-O-H)	Carbohydrates (Almeida et al., 2010)
1387	δCH_2_ Bending	Aliphatics (Yu et al., 2007)
1443-1446	δ(CH_2_)+δ(CH_3_)	Aliphatics (Yu et al., 2007)
1515-1535	-C=C- (in plane)	Carotenoids (Rys et al., 2014; Adar, 2017; Devitt et al., 2018)
1606-1632	ν(C-C) Aromatic ring+σ(CH)	Phenylpropanoids (Agarwal, 2006; Kang et al., 2016)
1654-1660	-C=C-, C=O Stretching, amide I	Unsaturated fatty acids (Jamieson et al., 2018), proteins (Devitt et al., 2018)
1682	COOH	Carboxylic acids (Sanchez et al., 2020c)
1748	C=O Stretching	Esters, aldehydes, carboxylic acids and ketones (Colthup et al., 1990)

**Table 2:** Vibrational bands and their assignments for the IR spectra collected from plant leaves and seeds.

Wavenumber (cm^-1^)	Vibration	Assignment
668	Out of plane ring bending	Aromatic ring (Colthup et al., 1990)
720	CH_2_ in-phase rocking	Alkanes (Colthup et al., 1990)
730	CH_2_ in-phase rocking	Alkanes (Colthup et al., 1990)
890	CH_2_ wag	Alkanes (Colthup et al., 1990)
947	C-O Stretch	Carbohydrates (Colthup et al., 1990)
958	C-O Stretching	Carbohydrates (Colthup et al., 1990)
1027	C-O Stretching	Alcohols (Colthup et al., 1990)
1093	Substituted Benzene	Aromatic ring (Colthup et al., 1990)
1155	C-O Stretching	Alcohol (Colthup et al., 1990)
1292	CH_2_ Twisting	Alkane (Colthup et al., 1990)
1305	C-O stretching	Alcohol (Colthup et al., 1990)
1378	CH_3_ Symmetric Deformation; OH deformation of carboxyl monomer	Alkane or carboxylic acid (Colthup et al., 1990)
1438	CH_2_ Stretching	Alkane (Colthup et al., 1990)
1463	CH_2_ Scissoring	Alkane (Colthup et al., 1990)
1472	CH_2_ Scissoring	Alkane (Colthup et al., 1990)
1696	C=O Stretching	Carbonyl compound (Colthup et al., 1990)
1710	C=O Stretching	Carbonyl compound (Colthup et al., 1990)

In the IR spectra, a vast majority of vibrational bands originates from CH_2_ vibrations of alkanes. Such spectra also possess the vibrational bands that can be assigned to carbonyl-containing compounds, such as acids, aldehydes, ketones and esters, as well as C-O vibrations that originate from carbohydrates. In our previous study, we demonstrated that IR and RS are complementary techniques in the characterization of the plant materials. For instance, IR spectra acquired from plant wax were highly rich with C-O-C, C-O-H and C=O vibrations that could be assigned to carbohydrates, alcohols, aliphatic and aromatic acids, aldehydes, ketones and esters. However, Raman spectra acquired from the same plant material only exhibited C-C, C-H and CH_2_ vibrations. Theoretical calculations revealed that with an increase in the length of carbon chain, the intensity of CH_2_ vibrations in Raman spectra increases in the fourth power, whereas only a linear increase was expected in the corresponding IR spectra. Since a vast majority of alcohols, acids, aldehydes, ketones and esters in plants have more than C18 carbon atoms, the intense CH_2_ vibrations obscure the appearance of other vibrational bands in the Raman spectra acquired from such materials. At the same time, theoretical calculations revealed that RS could be used to reveal conformations of aliphatic chains in such molecules that was not accessible by the IR spectroscopy.

## 3 Recent literature

### 3.1 Applications of Raman spectroscopy

#### 3.1.1 Phytopathology

Phytopathogens pose a significant threat to crops worldwide, with reports indicating that 10% to 40% of crops are lost annually due to these pathogens (Ristaino et al., 2021). Climate change exacerbates this issue, enabling the spread of pests to previously unaffected regions (Bebber et al., 2013). Therefore, timely disease detection is crucial for farmers to mitigate losses. During the past decade, Raman spectroscopy (RS) emerged as a valuable tool for addressing this challenge, outperforming traditional methods like qPCR in direct costs and detection limits (Sanchez et al., 2020e). Previous studies have explored its effectiveness across crops and diseases, including Huanglongbing (HLB) in oranges, bacterial and viral infections in tomatoes, and various fungal diseases in corn (Egging et al., 2018; Sanchez et al., 2019a, Sanchez et al., 2020b). Recent studies have continued expanding the application of RS to various crops and pathogens, providing new insights into the molecular origins of Raman diagnostics.

**Figure 3:**
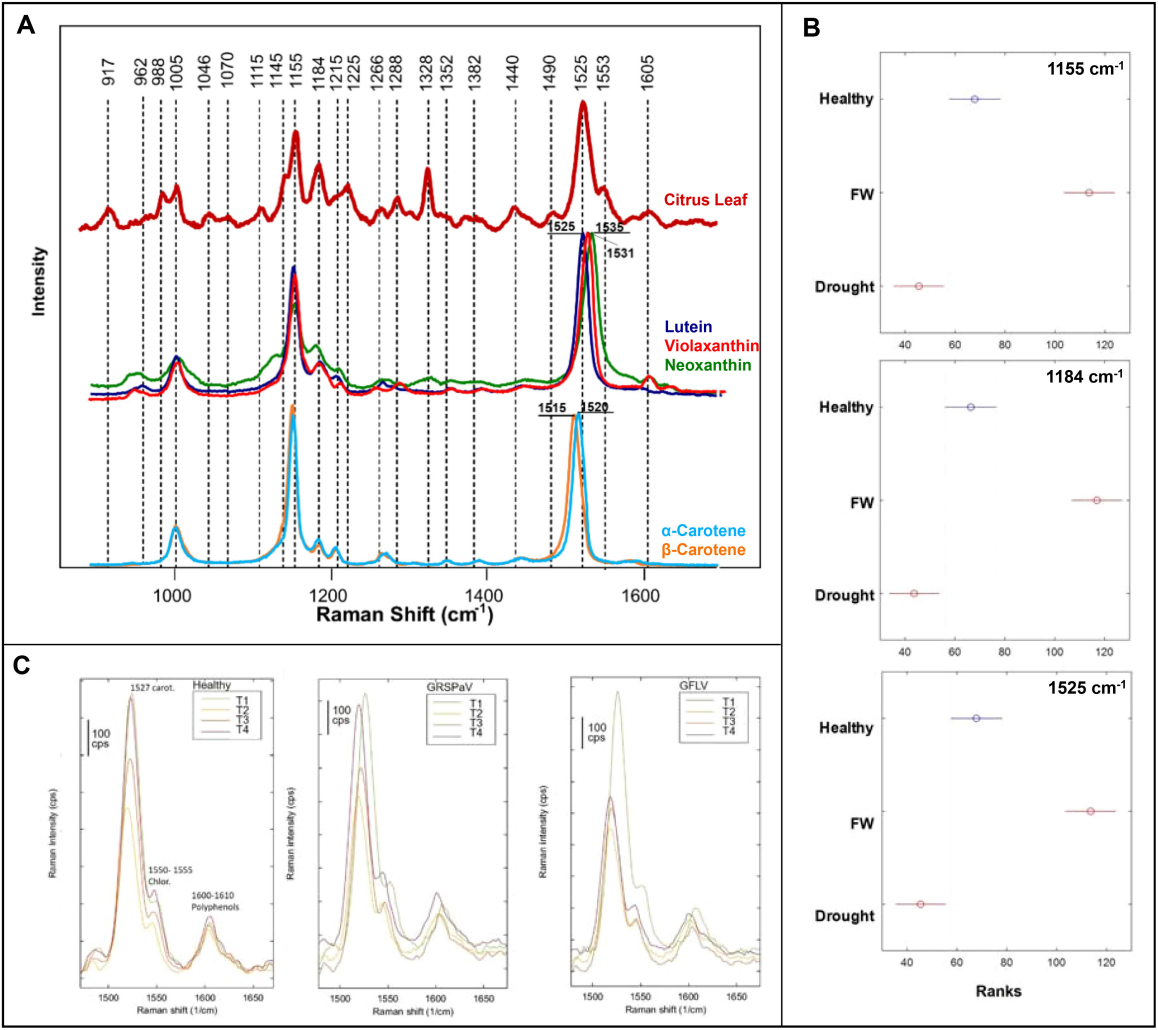
**(A)** Raman spectra of a citrus leaf compared to metabolite standards; **(B)** Kruskal-Wallis of Raman intensities at carotenoid peaks, comparing healthy banana leaves, fusarium wilt infected banana leaves, and drought stressed banana leaves; **(C)** Raman spectra of grapevine leaves at four timepoints (T1-T4) comparing the healthy crop with the two viral infections. Reproduced with permissions from (Dou et al., 2021; Mandrile et al., 2022; Parlamas et al., 2022).

HLB also known as citrus greening disease, is a devastating bacterial disease that is caused by *Candidatus Liberibacter*). HLB affects citrus production globally with no known cure and an annual cost estimated at $3.6 billion just in the US alone, early detection is critical (Ghosh et al., 2022). Previous studies by the Kurouski lab demonstrated RS was capable of early detection and differentiation of HLB from other biotic and abiotic stresses like nutrient deficiency and blight (Sanchez et al., 2019a, Sanchez et al., 2019a). To identify the underlying molecular nature of RS-based sensing of HLB, Dou and co-workers performed HPLC analysis of plant leaves collected from healthy and infected plants (Dou et al., 2021), **Figure 3A**. The researchers found drastic differences in the concentrations of several major carotenoids, including lutein, α and β-carotenes. Dou and co-workers also found a major decrease in the concentration of chlorophyll in the leaves of HLB-infected plants. Next, Raman spectra were acquired from the carotenoids identified by HPLC. It was found that spectroscopic signature of lutein matched the vibrational fingerprint observed in Raman spectra acquired form plant leaves. Based on these results, the researchers concluded that upon the spectroscopic analysis of plant leaves, RS primarily detected changes in lutein. This marks a breakthrough in connecting Raman’s diagnostic capabilities with traditional methods of phytopathogen diagnosis.

Fungi are a major crop threat, comprising the largest group of phytopathogens with nearly 8,000 species linked to plant disease (Hariharan and Prasannath, 2021). Notably, *Colletotrichum graminicola* causes anthracnose leaf blight and stalk rot in maize, costing the US economy nearly $420 million annually (Belisário et al., 2022). Farber and co-workers demonstrated that RS coupled with PLS-DA could diagnose stalk rot in maize at the early and late stages (Farber et al., 2021a). This conclusion was made based on the differences in the vibrational bands that could be assigned to cellulose, lignin, and carotenoids in the spectra acquired from healthy and infected plants. Furthermore, these spectroscopic changes correlated with lesion sizes in plant stalks. These results showed that RS could be used to predict cultivar resistance to *C. graminicola*. These experiments were performed with a handheld spectrophotometer equipped with an 830 nm laser. *Fusarium oxysporum*, is another fungus that causes fusarium wilt in bananas (Ploetz, 2021). Parlamas and co-workers were able to detect and identify tropical race 4 strain of *F. oxysporum* infection using RS and PLS-DA (Parlamas et al., 2022). This technique detected the fungal disease in bananas 40 days post-inoculation due to changes in carotenoid-associated vibrations, **Figure 3B**. They also showcased its robustness in distinguishing from drought stress. Both studies highlight RS’s relevance in addressing key fungal pathogens with significant economic implications in global agriculture.

Viruses, with their high mutation rates, present a critical challenge to traditional crop disease management. Rapid identification of infected crops is key, and RS can play a vital role in this solution (Rubio et al., 2020). Using RS coupled with PCA and PLS-DA, Mandrile and co-workers were able to detect grapevine rupestris stem pitting-associated virus and grapevine fanleaf virus in grapevine, a crop central to the economies of several southern European countries (Mandrile et al., 2022). Spectral changes, mainly in carotenoid content, were confirmed through quantitative chemical analysis, **Figure 3C**. Combining this with expression levels of enzymes involved in the carotenoid pathway, the researchers revealed that RS was detecting a metabolic cascade leading to the accumulation of abscisic acid and strigalactones. The study employed a dispersive Raman spectrophotometer equipped with a 785 nm laser. Importantly, this study connected enzyme expression data with changes within alterations observed Raman spectra.

Across bacteria, fungi, and viruses, RS consistently relies on changes in carotenoid quantity to differentiate diseased crops from healthy ones. These studies not only broaden the scope of crops and viruses tested but also enhance our understanding of the activated metabolic pathways during pathogenic stress and the specific metabolites responsible for Raman spectral changes.

#### 3.1.2 Environmental stress

Climate change’s escalating environmental disasters, coupled with on-going arable farmland losses and population growth, pose the greatest threat to food supply (Zandalinas et al., 2021). Drought is already the leading cause of agricultural loss, incurring approximately $37 billion in annual losses (Fao, 2018). Before 2020, early papers on RS’s application to abiotic stresses were limited, but recent advancements demonstrate its effectiveness in studying both environmental and anthropogenic stressors like pesticides and microplastics. This intersection of environmental toxicology and agriculture is critical, given crops’ ability to bioaccumulate harmful elements and compounds from the soil.

**Figure 4:**
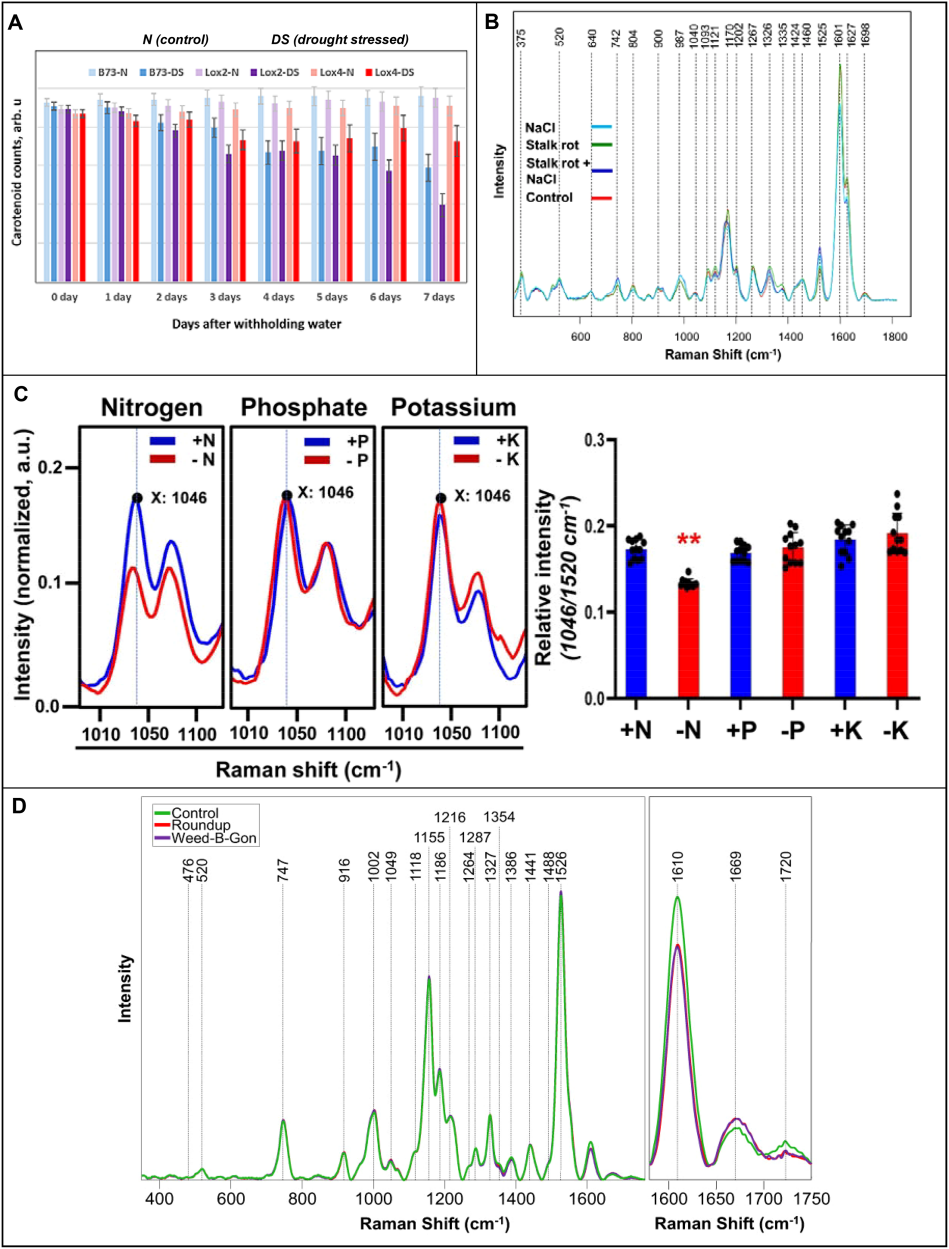
**(A)** Raman spectra of corn stalks exposed to biotic and abiotic stress at experimental day 2; **(B)** Histogram of carotenoid content in drought stressed and control maize lines; **(C)** Raman spectra of Arabidopsis leaves under macronutrient deficiencies with the corresponding histogram on the right; **(D)** Raman spectra of rose leaves one day after herbicide application. Reproduced with permissions from (Huang et al., 2020; Altangerel et al., 2021; Farber et al., 2023; Higgins et al., 2023).

Breeding resistant crop cultivars is a key strategy against environmental stressors. Altangerel and co-workers demonstrated that RS could assess drought tolerance in maize by studying carotenoid degradation (Altangerel et al., 2021). The researchers also found that only chloroplast carotenoids were depleted during osmotic stress. Finally, Altangerel and co-workers showed that RS could distinguish drought-resistant phenotypes within two weeks in seedlings by tracking carotenoid degradation, greatly expediting the development and testing of new maize cultivars, **Figure 4A**. This study was performed using a confocal Raman spectrophotometer equipped with a 532 nm laser. Similar breakthroughs were achieved by Higgins and co-workers that used RS to differentiate biotic and abiotic stresses in wheat and maize (Higgins et al., 2022b, Higgins et al., 2023). The first study focused on the analysis of wheat responses to nitrogen deficiency, drought, aphid infestation, and combined viral infections. Despite morphologically similar symptoms, RS coupled with PLS-DA successfully distinguished all stresses with above 90% accuracy. It has been also shown that changes in the Raman spectra (1185 cm^-1^) have direct relationship with the changes in the concentration of lutein, as was revealed by HPLC. The second study, Higgins and co-workers explored RS’s ability to identify individual and co-occurring stresses caused by salinity stress and stalk rot disease in maize, **Figure 4B**. It has been shown that RS was able to differentiate between individual and combined biotic and abiotic stresses in maize with greater than 90% accuracy. In both studies by Higgins and co-workers, the researchers utilized a handheld spectrophotometer equipped with an 830 nm laser. These studies contribute significantly to the application of RS in detecting diverse stress patterns and highlight RS’s ability to differentiate between biotic and abiotic stresses.

Nitrogen is a vital plant nutrient as a major chlorophyll component, and its deficiency significantly reduces plant productivity. While aerial imaging may be used to diagnose nitrogen deficiency, RS is more advantageous in distinguishing the condition from other causes of chlorosis (Mu and Chen, 2021). The Ram lab investigated the accuracy of RS in diagnostics of nitrogen deficiency in three different plant species: Arabidopsis, bok choy, and choy sum (Huang et al., 2020). The researchers found that nitrogen deficiency could be diagnosed by a decrease in the 1046 nm^-1^ peak, **Figure 4C**. It was also shown that changes in this band were not affected by phosphorus or potassium deficiencies. The second study validated these findings *in situ*, demonstrating the efficacy of the hand-held Raman device for field settings, while expanding to a wider range of crops (Gupta et al., 2020). Both studies used an 830 nm laser, with the first study employing a benchtop Raman spectrophotometer while the second employed a portable Raman clip. Besides environmental conditions, other abiotic stresses result from anthropogenic impacts on the environment.

Pesticides are considered indispensable in modern agriculture. However, the process of bioaccumulation, where plants absorb pesticides from the soil, poses a significant health threat to both humans and livestock. This is attributed to the highly toxic nature of many routine pesticides, even at minute concentrations (Li and Ai, 2023). In a recent study, Sanaeifar and colleagues used RS and electronic nose to assess the concentration of chlorpyrifos residues on tea leaves (Sanaeifar et al., 2021). The researchers employed artificial neural networks to construct a predictive model with a calibration curve measuring residues up to 0.35 mg/kg. The developed model relied on peaks associated with chlorpyrifos moieties at 631, 678, and 1240 cm^-1^. The study utilized a confocal Raman spectrophotometer equipped with a 532 nm laser. Herbicides also have toxic effects on crops and ornamental plants, as demonstrated by Farber and co-workers. In the recently reported study, RS was used to detect stress in roses induced by common lawn herbicides (Roundup and Weed-B-Gon) with 90% accuracy just a day after application (Farber et al., 2023), **Figure 4D**. The constructed PLS-DA model relied on major changes at the 1610, 1669, and 1720 cm^-1^ peaks, corresponding to phenylpropanoids, proteins, and carboxyl containing compounds. The study employed a handheld spectrophotometer equipped with an 830 nm laser. These studies, while proof-of-concept, showcase promising techniques applicable to a wide range of pesticides, emphasizing their potential impact.

Plastic pollution is an ongoing environmental and agricultural challenge. As large plastic pieces degrade into microplastics (smaller than 5 microns) and even smaller nanoplastics (less than 1 micron), these particles can be absorbed by crops, contributing to our eventual consumption of microplastics (Programme, 2022). Tympa and co-workers demonstrated the use of RS to sense microplastics in radishes through the direct detection of plastic peaks (Tympa et al., 2021). The microplastics were detected using a confocal Raman spectrophotometer with a 532 nm laser. Since there are limited spectroscopic studies on microplastics in agriculture, this research lays a solid foundation for future studies.

The myriad of environmental factors affecting plant growth challenges any diagnostic methods developed, however, RS has proven versatile in diagnosing stress from climate, nutrients, and anthropogenic threats. These studies lay a robust foundation for exploring additional abiotic stresses and enhancing our understanding of RS’s molecular detection capabilities.

#### 3.1.3 Phenotyping

Phenotyping is the foundation of plant breeding; therefore, the agriculture sector’s progress relies on developing new cultivars (Costa et al., 2019). Traits are usually selected for reasons like stress resistance and optimized crop features. Spectroscopy, particularly RS, excels in high-throughput plant phenotyping, which has been a limitation of traditional methods. Previous applications of RS include assessing crop quality in tomatoes and olives (Muik et al., 2004; Nikbakht et al., 2011), identifying crop genotypes (Farber et al., 2020; Morey et al., 2020), and distinguishing plants like hemp from cannabis (Sanchez et al., 2020a, Sanchez et al., 2020c). While current studies have continued exploring these areas, novel applications of Raman mapping in plant science are at the forefront.

**Figure 5:**
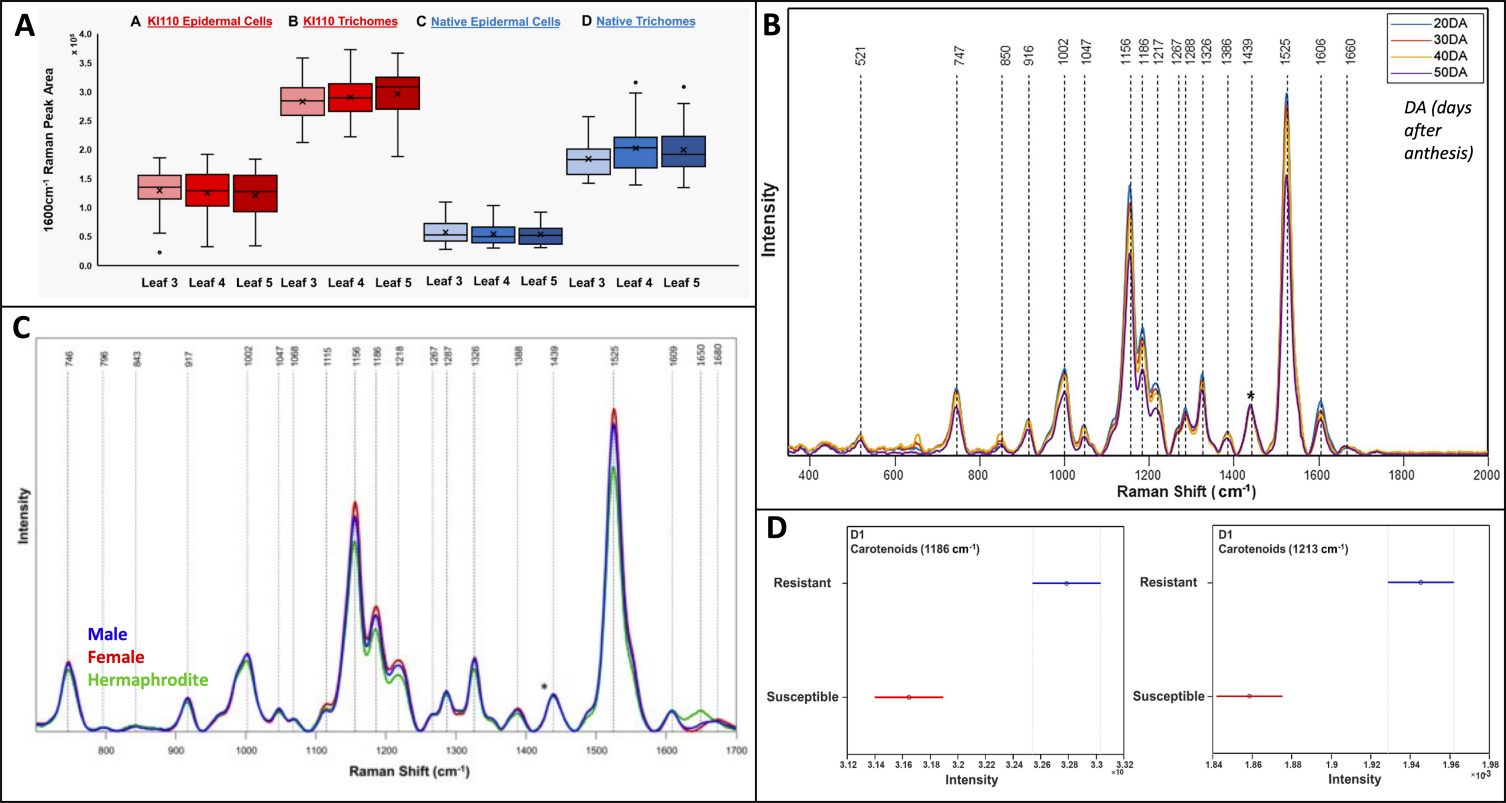
**(A)** Box and whiskers plot of rosmarinic acid abundance in spearmint comparing different leaf ages and structures for two cultivars; **(B)** Raman spectra of watermelon rind over maturation; **(C)** Raman spectra of mature cannabis plants comparing sex; **(D)** Raman spectra of phenotypically resistant and susceptible palmer amaranth one day after glyphosate exposure. Reproduced with permissions from (Li et al., 2021; Singh et al., 2021; Dhanani et al., 2022; Goff et al., 2022).

Accurate prediction of the optimal harvest date (OHD) is essential for successful crop harvesting (Xu et al., 2019). OHD relies on several metabolites such as phytochemical concentration, starch, and sugar content. While many crops show visual cues, vegetative crops often lack them, and certain fruits may not visibly indicate changes in sugar content. In addressing this challenge, Li and co-workers used RS to address this very problem in spearmint and found that the 1600 cm^-1^ peak had a linear increase with concentration of rosmarinic acid in the spearmint leaves as measured by HPLC (Li et al., 2021). The researchers also separated their spectra based on whether the portion of the leaf scanned contained trichomes or not, and how old the leaf was. Based on these results, the researchers found different leaf structures influence rosmarinic acid concentration, while leaf age did not, **Figure 5A**. Thus, these results showed that any leaf could be used for RS to determine OHD. In this study, the researchers used a confocal Raman spectrophotometer equipped with a 785 nm laser. Carotenoids, natural pigments responsible for vibrant colors in plants, exhibit varying concentrations that can either increase or decrease during ripening. As a proof of principle, Hara and co-workers optimized RS for the determination of carotenoid content in tomatoes, finding that for their specific system, 10 seconds of exposure correlated best with carotenoid concentration (Hara et al., 2021). In another study, Dhanani and co-workers used RS over a period of 50 days to track carotenoid concentration changes in watermelon rind to determine its ripeness (Dhanani et al., 2022), **Figure 5B**. The reported results demonstrated that RS could be used to determine a ripening stage of four different varieties of watermelons with around 85% accuracy. They also used resonant Raman to determine that lutein and β-carotene were the predominant molecular species in the rind responsible for the accuracy of RS-based sensing of fruit ripeness. Both studies used a handheld Raman spectrophotometer, with Hara using a 785 nm laser and Dhanani using an 830 nm laser. These detailed studies highlight RS’s robustness in providing precise information for harvesting decisions.

Most flowering plants have both male and female reproductive organs within each flower, but approximately 6% are dioecious, exclusively producing male or female gametes (Käfer et al., 2017). Dioecious plants are traditionally sexed through visual examination, which is extremely time-consuming. To this end, the Kurouski lab showed that RS could distinguish female hemp from male hemp. Specifically, Higgins and co-workers achieved over 90% accuracy in sex identification in both young and mature crops (Higgins et al., 2022a). Separately, Goff and co-workers used RS to differentiate dioecious from hermaphrodite hemp (Goff et al., 2022), **Figure 5C**. HPLC unveiled that RS distinguishes plants based on carotenoid concentration differences, with females having significantly higher lutein levels compared to both male and hermaphrodite hemp. Both studies employed an 830 nm laser-equipped handheld Raman spectrophotometer. These findings highlight RS’s future potential to automate sexing in hemp and other dioecious crops.

The fundamental science behind phenotyping is genotyping. While many physical traits are observable, some traits are not distinguishable like nutrient content and resistances. Furthermore, traditional genotyping with primers and qPCR can incur high costs. The Kurouski lab has applied RS as a cost-effective alternative in several crops. Notably, the researchers differentiated nutrient components in 15 rice genotypes using RS, identifying protein, polyphenol, and oil peaks for differentiation (Farber et al., 2021b). RS could also determine total starch content in rice grains. Genotyping experiments on peanut plants leaves achieved 94% accuracy for six genotypes when coupled with PLS-DA (Payne et al., 2022). Furthermore, the same model could identify resistance to nematodes with 83% accuracy. This study underscores RS’s potential in identifying multiple valuable crop traits after a model is trained on a sizable database. In another study, the Kurouski lab evaluated glyphosate-resistant Palmer amaranth (Singh et al., 2021), **Figure 5D**. After one day of exposure to glyphosate, RS could phenotype crops with 80% accuracy, while achieving around 70% accuracy for unexposed crops. All these studies used a handheld-Raman spectrophotometer equipped with an 830 nm laser. These studies have significantly advanced the field, particularly in seed genotyping. However, additional research is needed to fully assess the limits of RS in genotyping, particularly regarding resistance determination.

**Figure 6:**
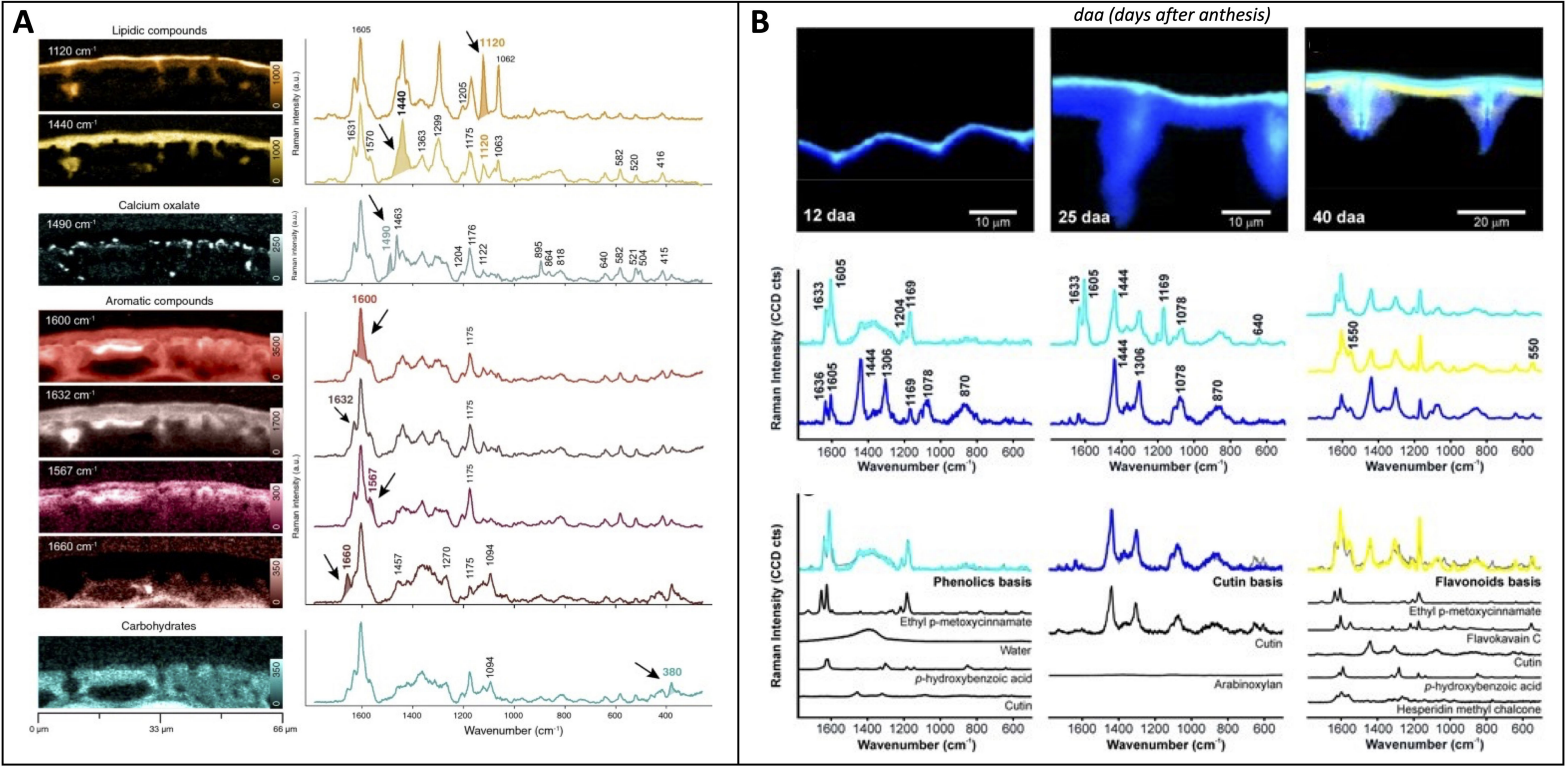
**(A)** Raman imaging of spruce needle visualized by the integration of various peaks corresponding to specific biomolecules; **(B)** Raman imaging visualized by unmixing analysis (NMF) of tomato cuticles from select stages of development, with the model fit spectra shown below the basis spectra. Reproduced with permissions from (Bock et al., 2021; González Moreno et al., 2023).

Raman mapping is a combined technique of RS and imaging where a laser is used across discrete section of an area, providing spatially resolved molecular information about the sample (Gordon and McGoverin, 2011). Images can be formed from the spectra based on the distribution of intensity for a selected spectral region. While not new in plant science, it has recently gained more exploration. The Gierlinger group broadened Raman mapping applications to various plants, particularly in recent projects focusing on imaging plant cuticles. These efforts bridged the gap between structural insights from electron microscopy and chemical analyses. In one study, the researchers used Raman mapping analyze cuticle layers in Arabidopsis stem, tomato peel, and spruce needle (Bock et al., 2021), **Figure 6A**. The images provided 300 nm spatial resolution on the composition of each layer, allowing the researchers to develop models of the cuticles. The researchers also determined molecular orientation using polarized laser light. Finally, they observed that higher concentrations of aromatic compounds resulted in better signal-to-noise ratios with a 785 nm laser compared to a 532 nm laser, albeit at the expense of spatial resolution. Another study by Gierlinger group aimed to comprehensively analyze the cuticle and epidermis of spruce needles (Sasani et al., 2021). Using their mapping technique, the researchers assessed several phytochemicals for changes in concentration and orientation across the cuticle. The researchers also used RS to track changes in tomato cuticle chemistry through maturity (González Moreno et al., 2023), **Figure 6B**. Specifically, the researchers identified variations in the concentrations of phenolics and flavonoids during ripening, enabling the construction of a novel model for cuticle development in tomatoes. This discovery revolutionized how we can study developmental plant biology. Separately, Zeng and co-workers used Raman mapping to visually quantify carotenoids and chlorophyll in tea leaves (Zeng et al., 2021). The researchers first used a confocal microscope to build a calibration model. This model was combined with map scanning spectra, allowing the researchers to predict analyte concentrations at any point on the Raman map. The study employed a 532 nm laser. These studies by the Gierlinger lab and Zeng et al. lay the groundwork for diverse applications of Raman mapping in plant science, paving the way for future endeavors.

The integration of RS into plant science has revolutionized phenotyping and genotyping, showcasing its versatility in agriculture. The recent applications emphasize the importance of carotenoids and aromatic molecules in Raman’s ability to distinguish crop traits. While applications such as sexing and mapping have limited current studies, further expansion in the scientific application of these underlying principles is anticipated.

### 3.2 Applications of surface-enhanced Raman spectroscopy

#### 3.2.1 Phytopathology and environmental stress

Traditional Raman in phytopathology has revolutionized proactive crop disease diagnosis by rapidly assessing plant metabolite concentrations. However, its diagnostic capabilities are limited by the magnitude of these changes. SERS offers solutions by detecting metabolites in minute quantities or detecting pathogens directly. Earlier SERS studies exemplify these strategies, albeit within a limited scope of research in plants (Kim et al., 2013; Lau et al., 2016; Zhou et al., 2020). Similar strategies could be used for detection of environmental stressors, as shown in a study tracking leaf-applied pesticide (Yang et al., 2017). Recent studies continue to expand on these SERS applications, exploring more phytopathogens and environmental factors.

**Figure 7:**
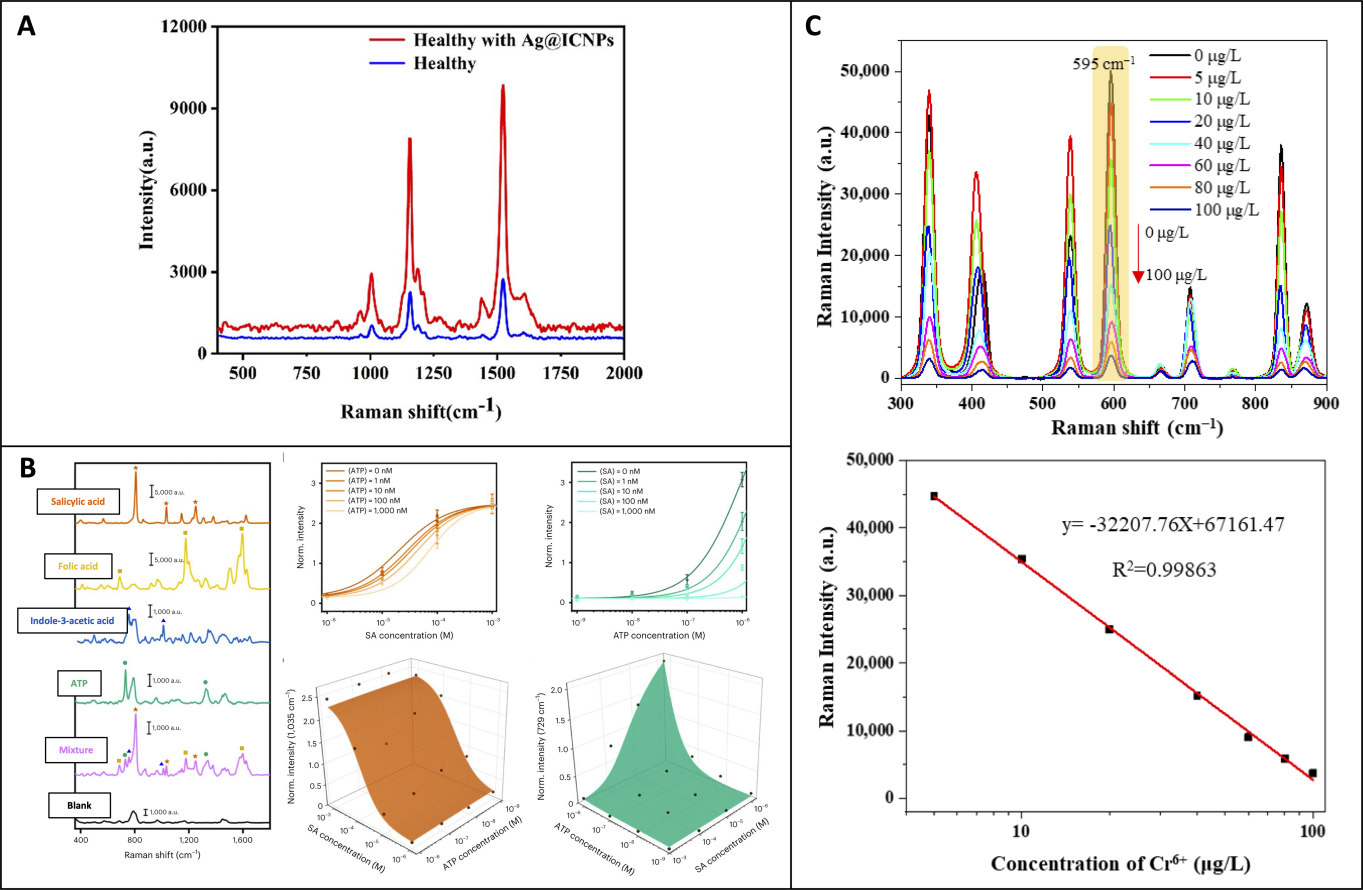
**(A)** Raman spectra of kiwifruit leaves with and without nanoparticles; **(B)** SERS spectra of various plant signaling molecules, along with concentration-dependence plots and three-dimensional plots of peak intensity at 1035 cm-1 and 729 cm^-1^ based on the combination ATP and salicylic acid concentrations. **(C)** SERS spectra of carbimazole at different concentrations of Cr6+, with a calibration curve below based on Raman intensity at the 595 cm-1 peak. Reproduced with permissions from (Jiang et al., 2023b; Son et al., 2023; Yin et al., 2023).

Direct pathogen detection offers advantages over traditional spectroscopy, overcoming challenges posed by pathogens’ evolved defenses that can destroy immune signaling molecules or evade detection by immune cells. This is crucial as these defenses often mask typical infection symptoms detected by Raman spectroscopy. Jiang and co-workers recently applied SERS to detect minute changes in carotenoid concentration in kiwifruit leaves caused by *Pseudomonas syringae*, the bacterium responsible for kiwifruit canker (Jiang et al., 2023b), **Figure 7A**. The study demonstrated SERS’s effectiveness in greatly amplifying signals from carotenoids compared to normal RS, both in early and late stages of disease. The researchers utilized silver nanoparticles coated with iodide and calcium ions and a confocal Raman spectrophotometer equipped with a 532 nm laser. Given the nanoparticles’ high specificity, one may question the need for individually designed nanoparticles for each specific analyte. A groundbreaking study by Son and co-workers showed that their nanoparticles could detect critical phytochemicals responsible for stress responses in watercress, wheat, and barley (Son et al., 2023). The researchers tracked changes in salicylic acid, ATP, phytoalexins, and glutathione, **Figure 7B**. These molecules exhibited unique vibrational bands that allowed the researchers to observe biotic factors (fusarium graminarium) and abiotic factors (wound stress, cold stress). The researchers also revealed that nanoparticles larger than 100 nm localized in the intercellular space without causing adverse effects. The study used silver-capped silicon nanoparticles coated in poly(diallyl dimethylammonium chloride) and a confocal Raman spectrophotometer equipped with 660 nm and 785 nm lasers. This research emphasizes that diverse nanoparticle development may not be necessary, as a single nanoparticle can effectively detect a wide range of conditions.

High-performance liquid chromatography is commonly used to monitor pesticide bioaccumulation in crops, but real-time tracking is challenging due to its destructive nature and lack of structural uptake information (Sicbaldi et al., 1997). Yang and co-workers demonstrated SERS could be used to track the uptake of the pesticide thiabendazole in tomato plants (Yang et al., 2019). The researchers observed a concentration-dependent journey of the pesticide from the midrib to the leaf margin when taken up by the roots. The nanoparticles detected thiabendazole directly, with the SERS limit of detection at around 2 µg/g of leaf tissue. The study used citrate-capped gold nanoparticles and a dispersive Raman spectrophotometer equipped with a 780 nm laser. Like pesticides, heavy metals are a significant focus of bioaccumulation in crops. Like pesticides, heavy metals bioaccumulate in crops due to naturally high levels in many agricultural regions worldwide. Yin and co-workers applied SERS to measure hexavalent chromium bioaccumulation in tea leaves (Yin et al., 2023), **Figure 7C**. They employed nanoparticles coated with methimazole, a compound which exhibits a selective reaction with chromium. Measurement of chromium concentration was achieved by tracking linear intensity decreases at the 595 cm^-1^ peak, which corresponded to decreases in methimazole concentration. This system achieved a limit of detection of 0.945 mg/kg of leaf tissue. The study utilized silver-coated gold nanoparticles capped with carbimazole, which hydrolyzes to methimazole, and a confocal Raman spectrophotometer equipped with a 785 nm laser. It’s worth noting that the study on thiabendazole was conducted *in vivo*, while the study on chromium used digested plant tissue. Both studies showcase diverse approaches to detecting minute toxic compounds in crops.

Whether by direct or indirect detection, there is major potential of SERS for highly specific detection of stress factors in crops in agriculture as researchers explore innovative detection methods. These SERS advancements hold promise for the future of crop disease diagnosis and environmental monitoring, offering several advantages over traditional Raman.

#### 3.2.2 Phenotyping

Similar chemical principles underlie the application of Raman and Surface-Enhanced Raman Spectroscopy (SERS) in phenotyping. However, a key distinction lies in SERS’s usage of custom nanoparticles for direct detection of specific proteins and DNA sequences (Almehmadi et al., 2019). This approach can resemble primer-based techniques such as qPCR, utilizing labels on nanoparticles; however, label-free detection of the nucleic acid itself is also possible.

**Figure 8:**
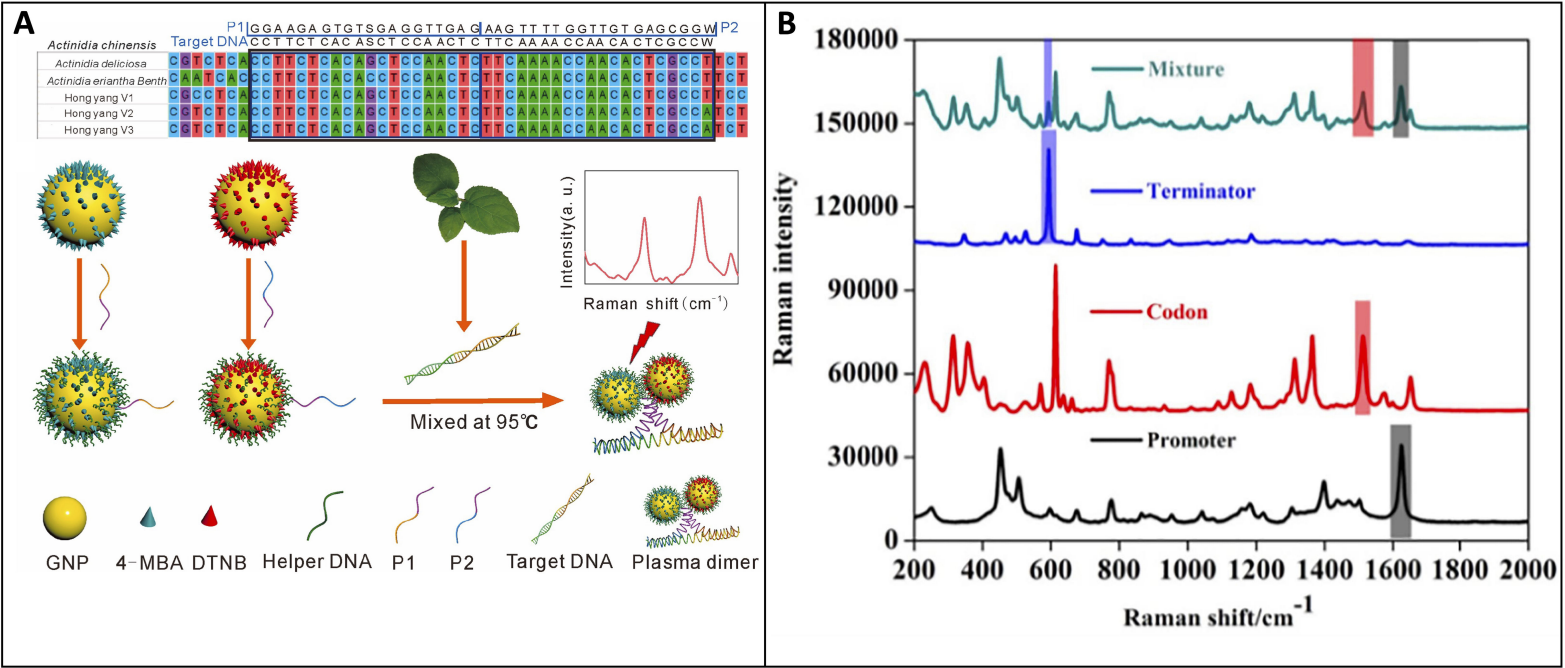
**(A)** The formation of GNPs combined with target DNA leading to plasmonic dimers with strong SERS enhancement of reporter molecules; **(B)** SERS spectra for three GMO components and differentiation within a mixture. Reproduced with permissions from (Yao et al., 2022; Jiang et al., 2023a).

SERS can overcome the challenges encountered by Raman and PCR in detecting nucleic acids, methods which are hindered by the low concentration of nucleic acids and the requirement for amplification. Dina and colleagues demonstrated that SERS could be used to amplify the intrinsic Raman signal of DNA without amplification, specifically focusing on potato and grapevine leaf tissue (Dina et al., 2022). The study utilized chloride-capped silver nanoparticles and a confocal Raman spectrophotometer with 532 and 785 nm lasers. This validates the feasibility of nucleic acid detection for plant tissue. Direct detection of specific DNA sequences has recently found success in sexing kiwi plants. Jiang and co-workers demonstrated that SERS could sex dioecious kiwi plants at an early age using gold nanoparticles coated in primer DNA and Raman reporters (Jiang et al., 2023a), **Figure 8A**. The researchers achieved a remarkably low limit of detection (100 fM) by employing two primers that bound to the male sex gene, vastly outperforming PCR. The resulting Raman signal could be used for sex assignment, relying on peak intensities at 1071 and 1326 cm^-1^ which corresponding to the two Raman reporters 4-MBA and DTNB. The study utilized a portable Raman spectrophotometer equipped with a 785 nm laser. Similarly, this technology finds application in detecting genetically modified organisms (GMO) components within crops. Yao and co-workers, for instance, utilized SERS in conjunction with a lateral flow strip to identify GMO components of soybeans (Yao et al., 2022), **Figure 8B**. Modified nanoparticles enabled the detection of three GMO components, and a linear correlation between SERS signal and analyte concentration was observed. This study utilized silver-shelled gold nanoparticles conjugated with thiolated DNA and Raman reporters. It also utilized a confocal Raman spectrophotometer. All studies were conducted using extractions and were therefore destructive, raising the future possibility of improving the technology for *in vivo* detection.

While conventional Raman excels in phenotyping, the low concentration of nucleic acids creates an opportunity for SERS to play a pivotal role. These recent studies highlight the potential of SERS as an invaluable technique for swiftly phenotyping crops, particularly when equipped with pre-prepared genetic segments of DNA.

#### 3.2.3 Other SERS-based studies

Recent SERS advancements in agriculture exhibit great potential yet pose questions about *in-vivo* nanoparticle use. Since engineered nanoparticles are meant to be absorbed by crops, they pose health risks for both plants and human consumers (Shrivastava et al., 2019). Variations in toxicity also complicate their applications; for instance, despite silver nanoparticles providing the best SERS enhancement, they also possess the greatest biological toxicity (Ferdous and Nemmar, 2020). Furthermore, nanoparticle translocation is not fully understood. Nanoparticles are primarily confined to intercellular spaces due to size limitations imposed by plant cell walls, so recent studies aim to understand nanoparticle move within plants and to improve their ability to infiltrate cells.

While studies have explored nanoparticle uptake, few have specifically tracked translocation with SERS. Yilmaz and team addressed this gap by exposing maize seedlings to silver nanoparticles and monitoring their translocation with RS (Yilmaz et al., 2021). The researchers observed accumulation in the root and the phloem of the stem, with smaller particle size linked to greater accumulation. Toxicity experiments revealed inhibition of root and leaf length, reduced chlorophyll content, increased protein content, and alterations in mineral composition. However, these effects will largely vary on the specifications of the nanoparticles. The study employed two sets of nanoparticles synthesized through chemical and green synthesis methods, utilizing a confocal Raman spectrophotometer equipped with a 785 nm laser. Size significantly influences the movement and effects of nanoparticles, and location-specific uptake is primarily restricted. For example, roots can take up nanoparticles smaller than 100 nm, leaves up to 50 nm, and cell walls generally limit cellular uptake to around 20 nm (Ballikaya et al., 2023; Wang et al., 2023). To solve this, Cupil-Garcia and co-workers designed 12 nm rod-shaped nanoparticles capable of penetrating past the tobacco leaf cell wall (Cupil-Garcia et al., 2023). Infiltration was verified with several techniques including, transmission electron microscopy, two-photon luminescence, photoacoustic imaging, and SERS. This multi-technique approach enables advancements in various spectroscopy fields. This study used silver coated gold nanorods and a confocal Raman spectrophotometer with a 785 nm. Importantly, successful nanoparticle infiltration into plant cells opens new possibilities for detecting intracellular proteins. There is still much to explore in the study of SERS nanoparticles, including their toxic effects and the development of new designs.

Advancing nanoparticle development for SERS applications in crops shows great promise. However, the utilization of nanoparticles in plants requires not only study of their photonic capabilities but also an assessment of the potential health risks they pose to both plants and humans. Still, as new nanoparticle designs emerge, the range of possible applications in agriculture will only continue to expand.

### 3.3 Other spectroscopic techniques

#### 3.3.1 Infrared spectroscopy

The complementary nature of Raman and infrared spectroscopies reveals similarities and differences in their capabilities for plant detection. Each technique exhibits preferences for specific moieties. Therefore, IR complements RS by potentially filling the detection gaps for compounds that Raman may miss. Previous applications of IR have successfully detected pesticides in cucumbers and diseases like zebra chip in potatoes and sour rot infection in tomatoes (Jamshidi et al., 2015; Liang et al., 2018; Skolik et al., 2019). The largest applications of IR in plant science however are phenotypic applications, such as studying fruit composition and structural characterization (Cozzolino, 2014; Farber et al., 2019). Recent studies have further expanded IR’s applications, investigating previously unstudied pesticides, other abiotic stresses, and continuing studies on plant composition.

In a study by Lu and co-workers, IR was applied to detect chlorpyrifos and carbendazim residues in cabbages (Lu et al., 2021). The researchers built chemometric models to quantify residue amounts in vegetables, allowing for the quick assessment of whether residues met food safety standards. The researchers noted lower decreased performance at trace pesticide levels, emphasizing the need for improved accuracy at lower detection limits. IR can also directly track metabolites, aiding in environmental stress detection. Zhang and co-workers used IR spectroscopy to identify drought stress in crops by measuring malondialdehyde content in pine needles (Zhang et al., 2021). The study extensively explored chemometrics, fine-tuning a PLS model for optimal prediction, ultimately achieving a model with an R^2^ value of 0.66. Drought stress is challenging to model due to co-occurrence with effects like high temperature and low humidity, therefore one can expect other modeled stresses to have greater viability. These studies both emphasize the role data manipulation has in predictively using IR spectroscopy.

**Figure 9:**
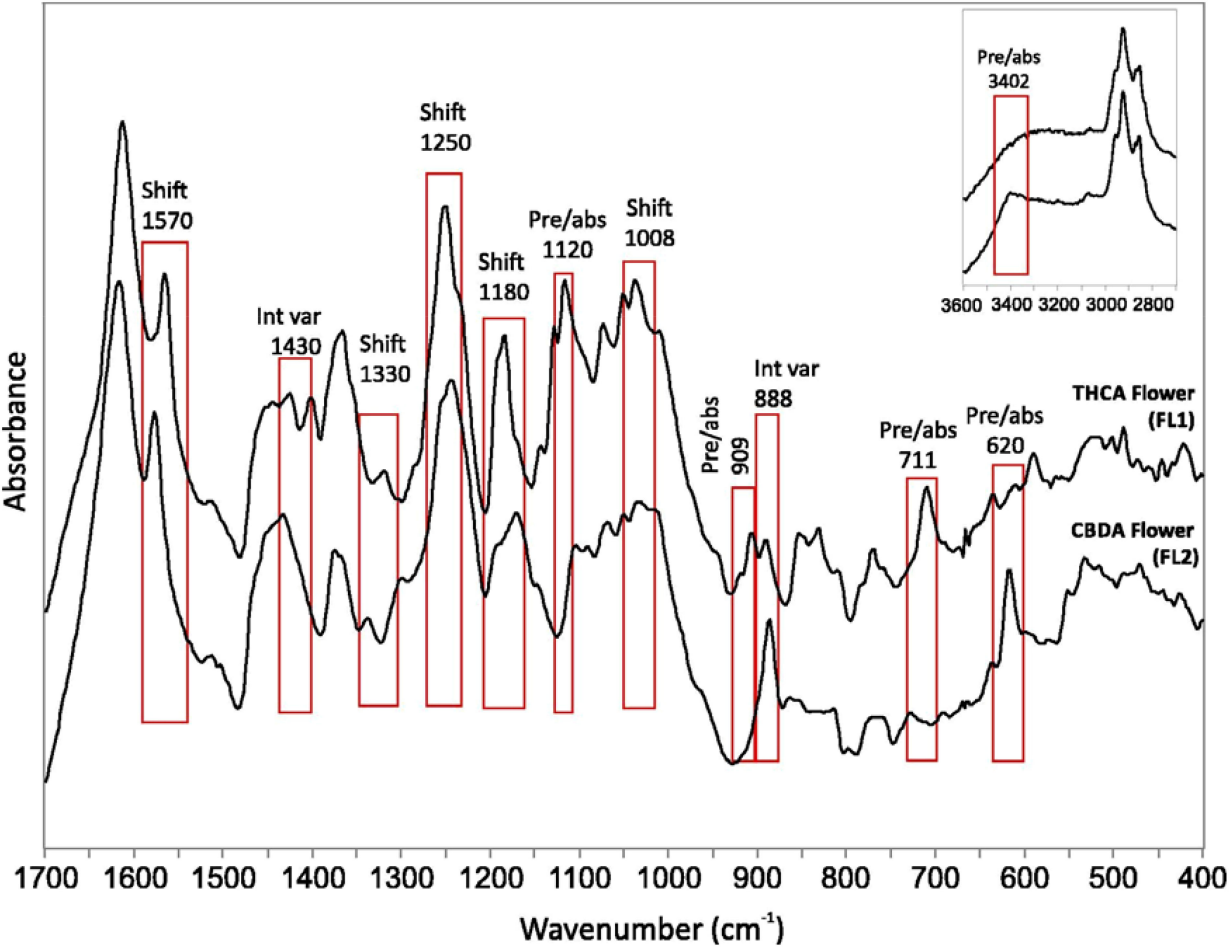
FTIR spectra of THCA and CBDA dominant flowers with major differences marked by rectangles. Reproduced with permissions from (Geskovski et al., 2021).

Determination of plant analytes is key for several sectors of the agriculture industry. Geskovski and co-workers used Mid-IR spectroscopy to quantify THC and CBD content in cannabis extract and flowers by constructing a multivariate model that achieved R^2^ values above 0.95 (Geskovski et al., 2021), **Figure 9**. The researchers also considered spectral differences between THC/CBD and their acidic precursor forms, emphasizing the importance of accounting for different metabolite forms in concentration determination. On the other hand, Miao and co-workers demonstrated that near-IR could be used for the accurate assessment of nitrogen content in rice, achieving an R^2^ value greater than 0.95 with synergy interval PLS (Miao et al., 2023). This was primarily based on the peak at 4854 cm^-1^, corresponding to the N-H stretch indicative of protein content. The study was conducted across various stages of crop growth, making the model relevant to all growth stages of rice. Although not applied to fresh plant tissue, the technique’s ease of use for high-throughput phenotyping remains a significant advantage. Understanding the structural composition of plant cell walls is crucial for comprehending plant development and biomass utilization (Pettolino et al., 2012). Liu and co-workers employed ATR-FTIR to characterize 58 cell wall polysaccharides, learning that individual peaks could determine cellulose, hemicellulose, and pectin content (Liu et al., 2021). The cell walls could then be differentiated based on the abundance of these polysaccharides. Still, vibrational similarities in molecules like arabinan and galactan limit the ability to distinguish cell walls that vary in concentrations of these two polysaccharides. Nevertheless, the study’s examination of numerous polysaccharides indicates strong potential for future applications in rapidly determining plant cell wall structure.

Many of these studies highlight the crucial role of chemometrics in distinguishing variations within the spectra. As models improve sensitivity, challenges like the strong signal from water molecules in IR should diminish. While Raman has seen more development in the plant field, there’s a significant need to explore diverse diagnostic and quantitative applications of IR to better understand its limits.

## 4 Current limitations and future perspectives

RS has rapidly expanded in agriculture, solving issues posed by traditional techniques. It has thoroughly been utilized for studying various phytopathogens (bacterial, fungal, or viral) and environmental stresses critical in our changing climate. RS excels in these aspects, marking a significant paradigm shift towards digital farming in agriculture. Many recent studies have predominantly focused on proof-of-principle experiments under controlled growing conditions, isolating a single variable. While these studies contribute to constructing stress response models, it is crucial to compare these models with actual field growing conditions. RS detects plant stress by monitoring changes in metabolite content, typically involving carotenoids and phenylpropanoids in most cases. However, as demonstrated by studies using RS to determine OHD, these metabolites can vary in concentration due to maturation. In addition, field conditions seldom involve isolated stresses, often featuring the co-occurrence of multiple minor stresses. Consequently, conducting more longitudinal studies on stress in actual field crops would bridge the gap between these controlled experiments and real-world application.

Phenotypic applications of RS further showcase the technique’s versatility, including determining OHD, crop sexing, and genotyping. The recent development of Raman mapping in agriculture is especially noteworthy. While offering limited advantages for real-time plant monitoring, it holds significant promise for the detailed examination of plant structure. Raman mapping has transformed human histological studies of diseases, an application which could be translated to plants. This could offer insights into how various adverse growth conditions and diseases impact plant histology and structure. Finally, while each study highlights the usefulness of RS for a specific aspect of a particular crop, there is a lack of comprehensive efforts to fully integrate RS for all aspects within a single crop. This end goal is to develop a complete chemometric model capable of predicting multiple stresses for a crop and monitoring its overall growth, marking the final step toward the widespread implementation of RS in daily agricultural practices.

As was mentioned in the introduction, substantial costs of Raman spectrometers limit broad utilization of RS in farming and plant breeding. With the current instrumental price, RS can be implemented as a service rather than a technology that can be possessed by every farmer or breeding center. Nevertheless, miniaturization of Raman spectrometers and reduction of their cost could be an avenue that will transform the use of this innovative technique in farming. Furthermore, it remains unclear whether spectroscopic library acquired at one geographic location could be directly transferred to detect plant stresses at other geographic locations. The same question can be posed about variability of signals from different varieties of plants. From one perspective, such specificity is advantageous to identify resistant and susceptible cultivars. However, from another perspective, it remains unclear whether detection and identification of plant stresses would require additional variety-specific calibration. Finally, RS-based assessment of plant stresses would benefit from coupling of this highly sensitive technique with approaches, such as RGB or thermography drone or satellite-based imaging. These imaging techniques could be used to detect ‘problem’ areas in fields that can be later inspected by RS to identify the problem.

SERS development in agriculture, though slower than RS, presents equally robust applications. Its strengths include detecting minute concentrations and specified targets, with most stress detection studies emphasizing the former. Carotenoid-linked peaks dominate the normal Raman spectra of plants, so SERS could feasibly distinguish a stress detectable through trace metabolites like ATP and salicylic acid. An overlooked application of SERS in agriculture is the direct detection of pathogens via antibody or DNA coated nanoparticles. This would offer a faster alternative to traditional methods like PCR for confirming disease. Despite its versatility, concerns about the impact nanoparticles have on plant and human health limit widespread application. Few SERS studies conduct toxicity assays that would build confidence in food safety. Additionally, the use of nanoparticles is step beyond base Raman, so studies using them must compare their application to base Raman to justify the use of SERS. Nevertheless, ongoing nanoparticle advancements will continue to incite novel applications for SERS in agriculture.

IR spectroscopy has found several applications in agriculture, although not as extensively as Raman. Its strength lies in identifying compounds that are not Raman active, making it valuable for analyzing plant matter compositions. However, the significant water signal detected by IR often restricts studies to extracts or dried plant tissue. This limits its field application and renders it an often-destructive technique. Despite these drawbacks, IR analysis of plant tissue remains faster than methods like HPLC or ICP-MS. While IR may face challenges in transitioning to digital farming, its usefulness in existing applications should not be overlooked.
